# Editorial: Micronutrients and metabolic diseases

**DOI:** 10.3389/fnut.2024.1380743

**Published:** 2024-03-13

**Authors:** Peng An, Yongting Luo, Aimin Yang, Jinhui Li

**Affiliations:** ^1^Department of Nutrition and Health, China Agricultural University, Beijing, China; ^2^Department of Medicine and Therapeutics, Prince of Wales Hospital, The Chinese University of Hong Kong, Shatin, Hong Kong SAR, China; ^3^Department of Urology, Stanford University Medical Center, Palo Alto, CA, United States

**Keywords:** micronutrient, vitamin, mineral, iron, phytochemical, polyphenol

Micronutrients, encompassing minerals, vitamins, and phytochemicals, actively involve in diverse metabolic processes, and play critical roles in the maintenance of the normal function of various systems within the body, including cardiovascular, digestive, immune, erythropoiesis, and bone health. A disruption or imbalanced intake of micronutrients will exert adverse impact on human health and potentially contributing to the development of metabolic diseases including but not limited to cardiovascular diseases (CVD), type 2 diabetes, and neurodegenerative diseases (1).

Dietary patterns such as Mediterranean diet and Dietary Approach to Stop Hypertension (DASH), characterized by their richness in antioxidant minerals, phytochemicals, vitamins, and unsaturated fatty acids that may improve cardiometabolic health, have been recommended as preventive or treatment approaches for metabolic diseases (2, 3), including cardiovascular disease and type 2 diabetes (4–6). Supplementation of certain micronutrient in at-risk populations has been proven to be a highly cost-effective intervention for improving metabolic diseases (1). However, the role of many micronutrients in metabolic diseases and the underlying regulatory mechanisms remains unclear. The underlying regulatory mechanisms governing the role of many micronutrients in metabolic diseases remain unclear, emphasizing the importance of continued investigation on the relationship in this field.

Research Topic “*Micronutrients and metabolic diseases*” comprises 22 articles covering the epidemiological and mechanistic studies investigating micronutrient metabolism and their influence on human health. Several articles in this Research Topic systematically evaluated the associations of micronutrients and cardiometabolic risk factors with risk of metabolic diseases. Zhang Y. et al. summarized the relationship among metal ion concentrations and multiple metabolic diseases based on recent studies using ionomic or multi-elemental profiling of different biological samples. Vahid et al. evaluated the association between micronutrient intake and obese/overweight in a case-control population consisting of 1,605 participants. Another prospective cross-sectional study reported that dietary magnesium and potassium intake were associated with lower body fat in 155 Chinese participants with impaired glucose tolerance (Chu et al.). Wan et al. investigated the associations between blood toxic and essential metals with blood lipids, and observed blood lead and blood magnesium concentrations were associated with the dyslipidemia in 998 participants living in Southern China. In a study involving 3,858 Chinese with papillary thyroid microcarcinoma, higher iodine intake was reported to be a risk factor for nodal metastasis (Zhao et al.). Additionally, a case-control study with 1,012 participants found that dietary intakes of calcium, magnesium, iron, zinc, and copper were inversely associated with the odds of glioma (Zhang W. et al.). These findings suggest that mineral status could serve as valuable indicators for the early detection and prognosis of some metabolic diseases, emphasizing the significance of maintaining adequate and balanced intake of certain micronutrient for overall human health.

For the role of a specific mineral, four articles discussed the role of iron in metabolic diseases. Sun et al. investigated the predictive value of iron-regulatory hormone hepcidin for iron-deficiency anemia risk during pregnancy in a prospective study of 353 Chinese women. Qiu et al. explored the causal association between systemic iron status and 24 specific mental disorders, revealing a detrimental effect of higher body iron stores on depression and psychogenic disorder. A mechanistic study by Bengson et al. dissected iron homeostasis in neural cells, and revealed that ferritinophagy could be implicated in the pathogenesis of neurodegenerative diseases. In the review article by Bao et al., the mechanistic connection between abnormal iron metabolism and osteoporosis induced by diabetes mellitus was discussed.

Vitamin D deficiency or insufficiency has been identified as risk factors for cardiometabolic diseases (7). In this Research Topic, three original studies investigated the associations of vitamin D with cardiometabolic risk factors and related diseases. In a prospective cohort study involving 1,926 individuals, blood vitamin D concentration displayed a non-linear association with risk of type 2 diabetes during a mean follow-up of 3 years (Hu et al.). A cross-sectional study conducted by Shan et al. assessed the association of vitamin D with metabolic syndrome and related risk factors in 1,505 female Chinese (Hu et al.). Chen et al. performed a mechanistic study to evaluate whether vitamin D deficiency during pregnancy could alter the metabolism of glucose and lipids in offspring. Vitamin D deficiency during pregnancy generated adverse effects on the metabolism of glucose and lipids in offspring. Notably, these adverse effects cannot be rescued via vitamin D supplementation after weaning, suggesting that maternal vitamin D deficiency may elevate the risk of metabolic disease for offspring in adulthood (Chen et al.).

Four articles in this Research Topic explored the cardiovascular benefits of phytochemicals. Guo et al. comprehensively reviewed the antioxidant, anti-inflammatory, anti-hypertensive, and lipid-lowering effects of tea polyphenols, as well as the underlying molecular mechanisms. In a meta-analysis of 39 randomized controlled trials by Micek et al., the impact of polyphenols from 100% fruit juices on cardiometabolic risk factors was assessed. They found that anthocyanins were shown to decrease total cholesterol and low-density lipoprotein cholesterol in a dose-response manner (Micek et al.). Another cross-sectional analysis of 12,424 adults from the United States National Health and Nutrition Examination Survey reported that serum carotenoid concentrations were inversely associated with the odds of cardiovascular diseases, especially heart attack and stroke (Wang et al.). Moreover, Zhu et al. conducted a functional study to improve the delivery efficiency of phytochemicals nuciferine and epigallocatechin-3-gallate via loading them into microgel. Oral administration of microgel containing these phytochemicals reduced serum lipids of rats receiving high-fat diet, likely by modulating key genes involved in lipid metabolism and improving the diversity of gut microbiota (Zhu et al.).

Despite of abovementioned works, articles in this Research Topic also explored the associations of vitamin B1 (Wen et al.), niacin (Paolini et al.), zinc (Mitchell et al.), and calcium (Chiu et al.) with cardiometabolic diseases. These articles provided novel epidemiological evidence on the impact of micronutrients on health outcomes and offer mechanistic insights into micronutrient metabolism. The collective findings hold promise for guiding the development of healthier dietary patterns and more effective strategies for the prevention of cardiometabolic diseases.

## Author contributions

PA: Writing – original draft, Writing – review & editing. YL: Writing – original draft, Writing – review & editing. AY: Writing – original draft, Writing – review & editing. JL: Writing – original draft, Writing – review & editing.
